# Regulation of Human Cytomegalovirus Transcription in Latency: Beyond the Major Immediate-Early Promoter

**DOI:** 10.3390/v5061395

**Published:** 2013-06-03

**Authors:** Matthew Reeves, John Sinclair

**Affiliations:** Department of Medicine, University of Cambridge, Addenbrooke’s Hospital, Hills Road, Cambridge, CB2 0QQ, UK; E-Mail: js152@cam.ac.uk

**Keywords:** cytomegalovirus, latency, gene expression, chromatin

## Abstract

Lytic infection of differentiated cell types with human cytomegalovirus (HCMV) results in the temporal expression of between 170–200 open reading frames (ORFs). A number of studies have demonstrated the temporal regulation of these ORFs and that this is orchestrated by both viral and cellular mechanisms associated with the co-ordinated recruitment of transcription complexes and, more recently, higher order chromatin structure. Importantly, HCMV, like all herpes viruses, establishes a lifelong latent infection of the host—one major site of latency being the undifferentiated haematopoietic progenitor cells resident in the bone marrow. Crucially, the establishment of latency is concomitant with the recruitment of cellular enzymes that promote extensive methylation of histones bound to the major immediate early promoter. As such, the repressive chromatin structure formed at the major immediate early promoter (MIEP) elicits inhibition of IE gene expression and is a major factor involved in maintenance of HCMV latency. However, it is becoming increasingly clear that a distinct subset of viral genes is also expressed during latency. In this review, we will discuss the mechanisms that control the expression of these latency-associated transcripts and illustrate that regulation of these latency-associated promoters is also subject to chromatin mediated regulation and that the instructive observations previously reported regarding the negative regulation of the MIEP during latency are paralleled in the regulation of latent gene expression.

## 1. Introduction

Human cytomegalovirus (HCMV) is an opportunistic pathogen that, like all herpes viruses, can establish a latent infection that persists for the lifetime of the host. In healthy individuals both primary infection and the reactivation of latent virus rarely causes any significant clinical symptoms due to a robust immune response in the host [1,2]. In contrast, infection or reactivation in immuno-suppressed transplant patients or immune-compromised late stage AIDS sufferers represents a major source of morbidity and mortality [3,4,5]. Furthermore, HCMV infection in utero remains the leading viral cause of infectious congenital disease [6]. As it is now clear that a major contribution to HCMV-mediated disease is due to the reactivation of latent virus, [2,4,7,8,9] a number of laboratories have studied intensively the mechanisms that activate and repress viral immediate early gene expression during lytic and latent infection, respectively.

## 2. Histones, Chromatin and Gene Expression

The ‘histone code hypothesis’ predicts that the signature of the post-translational modifications of histone proteins directly impacts on the transcriptional activity of the cell [10,11,12]. In the broadest terms, a promoter can be transcriptionally active or transcriptionally silent and this is influenced principally by the acetylation and methylation states of its associated histones [13,14,15]. As such, pan acetylation of histones H3 and H4 is directly linked with a promoter that is capable of transcription [14]. In contrast, trimethlyation at lysine residues 9 and 27 on histone H3 and the subsequent recruitment of heterochromatin protein 1 (HP1) or polycomb proteins, respectively, is indicative of a transcriptionally repressed promoter [16,17,18,19]. As with all biological systems caveats do exist. For instance, histone methylation is not exclusively indicative of repression—dimethylation at lysine residue 4 on histone H3 is a marker of a promoter that has been recently active [20]. Additionally, histone phosphorylation, which occurs at serine (10 & 28) and threonine (11 & 29) residues in the N terminal of histone H3, has been linked with both transcriptional activation and repression of promoter activity depending on the phase of the cell cycle [21].

The regulation of the major immediate early promoter (MIEP) during latency and reactivation has been the subject of a number of recent reviews and, thus, it will not be covered extensively, here. Suffice to say, the regulation of the MIEP during latency and reactivation exhibits many hallmarks associated with chromatin-mediated regulation of eukaryotic gene expression. The MIEP in latently infected CD34+ haematopoietic cells or circulating monocytes isolated from healthy seropositive donors is predominantly associated with HP1 [22]—a marker of a transcriptionally silenced promoter that is recruited to promoters via an interaction with histone H3 trimethylated on lysine 9 (H3K9) [19]. However, differentiation to a mature dendritic cell (DC) phenotype is concomitant with high levels of acetylation of the histones bound to the MIEP and a lack of HP1 binding [22]. Importantly, the differences in the pattern of the modifications of the histones bound to the MIEP in undifferentiated or differentiated myeloid cells correlates directly with the ability to detect of IE gene expression in these different cell types in healthy individuals [22,23,24,25,26].

It is now well accepted that differentiation-dependent changes in the post-translational modifications of histones around the MIEP regulate latency-associated repression and reactivation of MIEP activity. However, much less is known about viral gene expression associated with latent infection and whether this is also regulated by histone modifications. With the advent of more sensitive high throughput screening techniques, the identification of a small number of latency associated transcripts (which, in most cases, are also expressed during lytic infection) has been possible. These transcripts expressed during latent infection are from viral genes dispersed throughout the viral genome, suggesting that no one region is particularly ‘latently active’ but rather that latency-associated transcripts are regulated independently by specific promoters.

In this short review, we will present the current status of our knowledge regarding the regulation of expression of the latency associated transcripts of HCMV and its relationship to post-translational modifications of histones.

## 3. Human Cytomegalovirus Latent Gene Products

A key biological property of all herpes viruses is their ability to undergo latent infection during which time only a subset of viral genes are expressed. Latent herpes simplex virus (HSV) gene expression is predominantly restricted to one locus—the latency associated transcript (LAT) region [27]. Alternate splicing generates multiple non-coding RNAs with functions ranging from anti-apoptosis [28], anti-sense mediated inhibition of lytic gene expression [27,29] and the generation of miRNAs that influence gene expression during latency [30]. In contrast, a number of patterns of Epstein-Barr virus (EBV) latent gene expression have been reported based on the phenotype of the infected lymphocyte [31]. The initial infection and transformation of a resting B cell (latency III) is concomitant with the expression of a number of gene products including EBNA-1, -2, 3A-C, EBNA-LP, LMP-1, 2A and B, as well as the untranslated EBER transcripts. It is hypothesised, based on studies of cell lines and the analysis of tissue ex vivo that the level of EBV latent gene expression is down-regulated (latency II - EBNA-1, LMP-1, LMP-2A and the EBERs) with the LMP proteins thought to drive the differentiation into latently infected memory B cells [32,33,34,35,36]. Consistent with this, circulating immunoblastic lymphomas (latency III) are rarely detected *in vivo* likely due to the robust immune response directed against EBV in the host promoting their elimination. Indeed, the most common phenotype (latency 0) is genome carriage in the absence of latent gene expression [37]—although LMP-2A transcripts have been detected in these cells [38]. However, dividing memory B cells that display a latency I phenotype (EBNA-1) have also been detected *in vivo* [35].

In contrast to HSV and EBV, the analysis of latent HCMV gene expression is in its relative infancy. Latent transcripts arising from the major immediate early region had been identified in specific populations of granulocyte-macrophage progenitors by the Mocarski laboratory in the 1990s [39,40] and US28 transcripts were also reported in an experimentally infected THP1 leukaemia cell line [41], but the application of high throughput molecular approaches led to the identification of a number of novel latent transcripts. Microarray technology employed by the Shenk and Slobedman laboratories detected transcription from a number of different loci in experimentally latently infected myeloid progenitor cells [42,43]. Subsequent work confirmed that some of these products were also expressed during natural latency and included UL138 [44], UL111A [43] as well as the UL81-82 anti-sense transcript (UL81-82ast) [45,46]. Although it is worth noting that the latent transcriptomes reported by these studies are not entirely overlapping, likely due to different cell types and viral strains used in the analyses. Indeed, the possible effects of using different viral strains is no better illustrated than by recent work showing that the expression of UL144 during latency appears to be dependent on the strain of virus used [47] and is discussed in more detail later.

The selective expression of viral genes during latent infection is likely to depend on similar mechanisms known to modulate the MIEP during latent and lytic infection. Importantly, aberrant expression of viral gene products not required during latency would likely risk triggering a robust host immune response, from the high memory T cell population known to recognise lytic antigens in normal HCMV carriers, with no benefit to the virus. Consequently, tight control of viral gene expression during latency is likely to be of some import. Since, most of the viral genes expressed during lytic infection are regulated by the prodigious activity of the IE72 and IE86 proteins [48,49,50,51,52], a failure to express a number of genes during latency can likely be attributed to the lack of IE expression resulting from extensive silencing of the MIEP which is clearly observed in latently infected myeloid progenitor cells (reviewed in [53]). Clearly then, gene expression during latency must be subject to mechanisms of regulation which are independent of the functions of viral IE gene products. In this review, we will use the regulation of two gene products—UL81-82ast and UL144—to illustrate potential mechanisms for the control of latent gene expression by HCMV in myeloid progenitor cells.

## 4. The UL81-82 Antisense Transcript—LUNA

Of all the putative latent transcripts, the factors controlling expression of the UL81-82 antisense transcript (UL81-82ast) during latent and lytic infection are arguably the best understood [46,47,54]. UL81-82ast was identified by Bego *et al.* [45] whilst searching for the UL81 transcription detected in the previous study by Goodrum *et al.* [42]. Bego *et al.* identified RNAs spanning the UL81 region in naturally latent monocytes and showed that transcription of this RNA actually occurs from the opposite strand encoding UL81 (hence the designation UL81-82ast), giving rise to a putative 133aa serine rich protein called LUNA (Latency Unique Nuclear Antigen). Although there is no known function attributed to LUNA during natural latency, there is some recent evidence that expression of LUNA may impact on HCMV carriage and reactivation *in vitro* [55].

Superficially, at least, the regulation of LUNA promoter activity during lytic [54] and latent [46,47] infection appears to be mediated by very different mechanisms. However, closer scrutiny reveals that a number of the key proteins involved in latent or lytic LUNA transcriptional regulation actually exhibit overlapping functions—this provides an interesting model for the complex regulation of herpes virus gene promoters during different phases of infection.

### 4.1. Regulation of LUNA during Latent Infection

After confirmation that the expression of LUNA could be detected in experimentally and naturally latent CD34+ cells, a very simple question was asked—is the LUNA promoter associated with histone proteins during latency and, if so, what post-translational modifications do these histones have? Chromatin Immunoprecipitation analyses of the LUNA promoter alongside the MIEP showed that the promoter is indeed associated with histones [46]. Furthermore, the LUNA promoter, in contrast to the MIEP, was associated with acetylated histones [22,46]. Thus, in the latent phase of infection, the LUNA promoter is associated with histone post-translational modifications that support gene expression.

### 4.2. Regulation of LUNA during Lytic Infection

**Figure 1:**
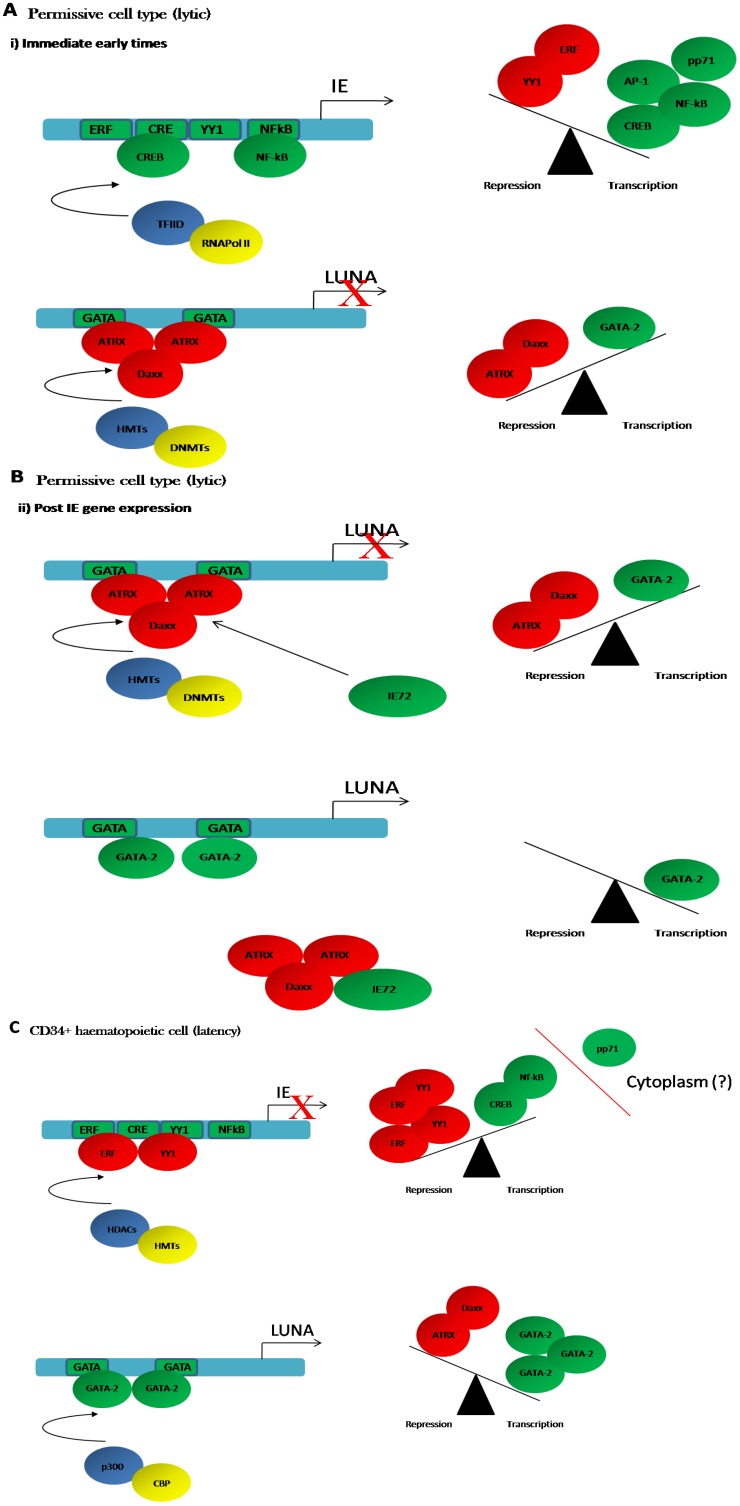
A model for the regulation of the major immediate early promoter (MIEP) and Latency Unique Nuclear Antigen (LUNA) promoter during lytic and latent infection. (**A**,**B**) Lower levels of GATA-2 expression in permissive differentiated cell types favour the recruitment of ATRX/Daxx to GATA-2 binding sites in the LUNA promoter to establish a repressive phenotype. As the lytic infection proceeds (**B**), IE72 is expressed which sequesters ATRX/Daxx complexes thus removing them as binding competitors at the LUNA promoter. This potentially increased GATA-2 binding which would promote LUNA gene expression in differentiated cells; (**C**) Latency is established following the infection of non-permissive haematopoietic CD34+ cells. High levels of transcriptional repressors bind to the MIEP and promote the formation of a repressive chromatin structure via the recruitment of histone modifying enzymes (histone methyltransferases (HMT) and histone deacetylases (HDAC). The relatively lower availability of transcriptional activators is possibly exacerbated by the exclusion of the major MIEP transactivator, pp71, from the nucleus in CD34+ cells. In contrast, high levels of GATA-2 are present in CD34+ cells and thus promote LUNA gene expression. In lytic infection, the scales are tipped towards transcriptional activation of the MIEP by relatively higher levels of activators versus repressors and the co-operative activity of pp71.

Although classified as a latent transcript, LUNA, like other latency associated transcripts, is also expressed during lytic infection [45]. Broadly speaking, the regulation of viral gene expression during lytic infection is subject to regulation by histone proteins [56,57,58,59,60,61] and, consistent with this including LUNA, the transfected LUNA promoter is responsive to the histone deacetylase inhibitor, Trichostatin A (TSA). Furthermore, increased expression of LUNA was detectable in virally infected cells incubated with TSA [54]. Further investigation identified that the expression of IE72 was critical for LUNA expression during lytic infection. Interestingly, IE72 has been hypothesised to drive early and late viral gene expression via the sequestration of inhibitory histone deacetylase activity [59]. However, unlike reported for other IE72-responsive genes LUNA gene expression in an IE72 null background was not rescued by TSA alone [54] suggesting that, although histone proteins may play a role, additional regulatory mechanisms besides IE72-mediated sequestration of histone deacetylases were needed for LUNA promoter activation [54]. Further work showed that activation of the LUNA promoter during lytic infection involved IE72 overcoming the repressive activity of the cellular repressor hDaxx and its binding partner, ATRX [54] (Figure 1A,B). Both hDaxx and ATRX have also been shown by a number of groups to exert a profound phenotype on the activity of the MIEP and these cellular proteins are targeted by incoming tegument protein, pp71 [60,62,63,64,65].

These observations are consistent with the view that chromatin architecture, whilst providing the framework for controlling gene expression, is not the only factor involved in LUNA promoter regulation. Indeed, it is interesting to note that parallels occur in other herpes viruses. EBV encoded BNRF1 disrupts the hDaxx:ATRX interaction to relieve early gene expression from the transcriptional silencing imposed by this complex also [66].

### 4.3. LUNA Is Regulated by the GATA Transcription Factors during Latency

The obvious question that arises from these observations is what drives differential LUNA promoter activity during latent infection? During latency LUNA expression must be independent of IE72 (by definition) whereas LUNA gene expression during lytic infection is IE72-dependent. Clues to the regulation of the LUNA promoter during latency came from a bioinformatics approach identifying a number of putative transcription sites in the LUNA promoter, including the GATA family of transcription factors [47]. GATA transcription factors are expressed in the haematopoietic cell lineage and have differential expression profiles dependent on the cell fate [67]. GATA-2 is a transcription factor expressed in early myeloid progenitors and declines in levels as myeloid cells differentiate and is sometimes referred to as the ‘master regulator’ of haematopoietic progenitor cells [68,69]. Certainly, GATA-2 is essential for haematopoiesis providing credence for such a grandiose moniker [69]. Furthermore, a lack of GATA-2 results in a severe defect in the generation of the granulocyte/ macrophage progenitor population [68]—a cell type which represents an important site of HCMV latency in the myeloid lineage [24,39,70]. Pertinently, GATA-2, along with other family members, has been shown to interact with chromatin modifying enzymes [71,72]. Amongst these interaction partners are histone acetyltransferases (pCAF and CBP/p300) which are important for histone and non-histone protein acetylation, including the GATA proteins themselves [71,72,73]. Similarly, GATA proteins also interact with histone deacetylases (HDACs) with GATA-2 interacting with HDACs-3 and -5, but not HDAC-1 [74] and thus, like a number of proteins, GATA-2 can promote the deacetylation as well as acetylation of target proteins including itself.

Interestingly, the LUNA promoter is GATA-2 responsive in transfection assays [46,47] and we have observed that GATA-2 over expression can rescue LUNA gene expression in ∆IE72 virus infected cells (M.R. and J.S., unpublished observations). Furthermore, a study that assessed the impact of HCMV latency on the expression of cellular miRNAs identified that mir92a was down-regulated in latently infected cells [75]. Interestingly, a target of mir92a is GATA-2 and, consistent with this, GATA-2 expression levels were elevated in latently infected cells [75]. Thus, at least in latency, it can be argued that HCMV promotes a micro-environment that supports the expression of LUNA by modulating the availability of the GATA-2 transcription factor. Taken together, these observations argue that during latency high levels of GATA-2 bind to the LUNA promoter and propagate a chromatin environment that is pro-transcriptional. However, during lytic infection, depletion of hDaxx:ATRX complex is required for LUNA gene expression to occur [54]. A pertinent question is why, then, does this complex not repress LUNA promoter activity in myeloid progenitors? One possible explanation is that there are differences in the relative balance of transcriptional activators and repressors which determine the activity of the LUNA promoter in different cell types. The viral MIEP provides a paradigm for this type of differential regulation in undifferentiated and differentiated myeloid cells (Figure 1). High levels of transcriptional repressors that bind the MIEP are present in early myeloid progenitors (Figure 1C) [22,76,77,78,79,80]. Furthermore, HCMV binding and entry into undifferentiated myeloid cells has been suggested to up-regulate transcription factors that would promote an environment repressive for the MIEP [81]—an event that does not occur upon infection of permissive fibroblasts [82]. In contrast, myeloid cell differentiation is concomitant with changes in both the absolute levels of repressors as well as the activation of transcriptional activators of the MIEP [22,83,84,85]. In the context of the LUNA promoter, the hDaxx:ATRX complex is likely to be stable in many cell types. 

However, we hypothesise that in early myeloid cells high levels of GATA-2 (which would alone act as positive regulator of the LUNA promoter) out-compete the repressive hDaxx:ATRX complex (Figure 1C). In contrast, in more differentiated cells with functional levels of repressive hDaxx:ATRX, but lower levels of GATA-2, this GATA-2 mediated competition of hDaxx:ATRX repression would not occur (Figure 1B) and hDaxx:ATRX-mediated repression would need to be overcome by e.g., IE72 during lytic infection (Figure 1B). Consistent with this view, as stated above, over-expression of GATA-2 into ∆IE72 infected fibroblasts can drive LUNA gene expression. In essence, the low levels of GATA-2 in more differentiated cell types that HCMV lytically infects may result in the additional requirement of IE72 activity against hDaxx:ATRX to promote LUNA gene expression during lytic infection. Provocative evidence in support of the competition hypothesis is derived from a number of biological properties of ATRX. Firstly the N terminal region of ATRX encodes domains that bind to histones—particularly methylated histone H3 on lysine 9 [86] and, thus, associates with heterochromatic DNA where it establishes a functional interaction with the hDaxx protein [87] to promote transcriptional repression. Furthermore, in addition to encoding helicase and ATPase functions [88], the ATRX protein contains coiled-coiled regions that allow direct binding to DNA—with affinity for DNA regions encoding GATA protein binding sites [89] and thus could provide direct competition with GATA transcription factors for DNA promoter occupancy.

## 5. TNF Receptor Superfamily Member—UL144

The UL144 gene product was originally identified as an orthologue of the tumour necrosis factor receptor superfamily member, herpes virus entry mediator (HVEM) [90,91]. As such, UL144 like HVEM can interact with BTLA to inhibit the proliferation of activated T cells [92]. As well as this intercellular function, UL144 has been shown to modulate intracellular signalling to regulate further immune responses to infection by hijacking NF-kB signaling [93,94]. UL144 recruits cellular TRAF-6 to promote CCL22 expression [94]—a cytokine that has been shown to promote the migration of Th2 and T regulatory cells which could impact on immune-surveillance and clearance of infected cells by the Th1 T cell repertoire [95]. The activity of UL144 shares characteristics with the LMP2A gene product of EBV which is expressed in persistently infected B lymphocytes [32,35] and also results in increased CCL22 production [96]. Furthermore, although UL144 expression during latency was not identified in a previous study [42], the UL144-148 region of HCMV was shown to be important for more efficient establishment of latency in an *in vitro* system [44] and a subsequent re-investigation of UL144 expression during latent infection showed that UL144 was, indeed, expressed during HCMV latency [47].

### 5.1. UL144 Is Expressed during Latency in a Strain-Specific Manner

In lytic infection, the UL144 protein is expressed with early kinetics reaching steady state levels by 48 hours post infection [90]. Although suggestive that expression is dependent on IE gene expression during lytic infection, the first insights into the regulation of the UL144 promoter came from an analysis of different HCMV isolates during latent infection of myeloid progenitor cells and, interestingly, suggested some parallels to those observed with LUNA [47]. Analyses showed that UL144 expression did occur during latency but that this appeared to be isolate dependent [47]. Importantly, previous studies [42,43] that had failed to identify UL144 expression during latency were performed using strains of HCMV that were either UL144 deficient (AD169) or UL144 null for expression during latency expression (TB40/e and VR1814) - based on the these recent observations of Poole *et al.* [47]. In contrast, the HCMV sequence reference strain, Merlin [97], was UL144 positive for expression during latency. Thus the differences between these and previous data were easily reconciled and, furthermore, provided a level of cross validation for the respective studies. 

### 5.2. UL144 Expression during Latency Is Dependent on GATA-2 Binding Sites

The observation by Poole *et al.* [47] that all the HCMV isolates analysed routinely expressed UL144 during lytic infection, even though this was not the case for latent infection, suggested that differential expression of UL144 during latency was a function of UL144 promoter activity specifically in myeloid progenitor cells. Sequence analysis of UL144 promoters showed that, although a high level of conservation was evident, a UL144 positive phenotype during latency correlated with the presence of putative GATA-2 binding sites in the UL144 promoter. Consistent with this, UL144 reporter constructs based on the Merlin Ul144 promoter were GATA-2 responsive whereas TB40/e-based UL144 reporter constructs were not. Additionally, the deletion of the putative GATA-2 sequences was sufficient to render the Merlin UL144 promoter inactive [47].

Direct evidence for a physical interaction between GATA-2 and UL144 promoter correlating with gene expression was also obtained by performing chromatin immunoprecipitation assays on infected myeloid cells. Crucially, GATA-2 immuno-precipitation with the UL144 promoter was only observed in cells infected with strains of HCMV that supported UL144 expression [47]. Furthermore, GATA-2 binding correlated with the detection of histone H3 di-methylated at lysine 4 bound to the UL144 promoter. In themselves, these data argue that the UL144 promoter is regulated by post-translational histone modifications around the UL144 promoter during latent infection likely modulated by GATA-2. Pertinently, the parallels with the LUNA promoter are overt [46]. Although LUNA expression during latency is independent of viral strain used, the LUNA promoter binds GATA-2 and is also responsive to GATA-2 in transfection assays [47]. 

## 6. Concluding Remarks

It is becoming increasingly clear that the regulation of cytomegalovirus gene expression during all phases of lytic infection involves post-translational modification of histone proteins (reviewed in [98]). Work from a number of laboratories has now demonstrated quite clearly that extensive chromatinisation of HCMV genomes occur [56,57,58,60,61,99,100] and that, during lytic infection, at least, the virus has to modify chromatin to control viral gene expression [59,101]. What is now also becoming clearer is that a complex interplay between cellular transcription factors and higher order chromatin structure with viral promoters is equally as important for the latency-associated regulation of viral gene expression [22,46,102]. 

Although the exact order of events controlling viral gene expression is still not fully understood, a model for the control of latent viral gene expression, based on a number of instructive observations, can be made. Firstly, the viral genome in the virion is naked—no histone proteins can be detected [56,58] consistent with a failure to detect histone proteins in purified virions by mass spectrometry [103]. This argues that the incoming viral genomes are subject to cellular responses that promote chromatinisation of the viral genome. Whether this is true for all foreign DNA is unclear but the association of plasmids and non-viral vector delivered DNA with histone proteins is documented *in vitro* [104] and *in vivo* [105]. The association of herpesvirus genomes with ND10 bodies—sites of extensive accumulation of histone modifying enzymes in the cell—is intrinsically repressive (reviewed in [106]). Indeed, the concerted attempt made by HCMV (and other herpes viruses) to disrupt these structures suggests they are anti-viral (reviewed in [107]). Consistent with a global repression of viral gene expression immediately post infection, studies of histone proteins at low MOIs show that a substantial number of incoming viral genomes are associated with methylated histone proteins [56] and that this response could be mediated by the action of a number of cellular proteins (*i.e.*, hDaxx, PML and Sp100) that accumulated at ND10 bodies. Consequently, the virus, through the action of pp71 [62,63,65,108] and subsequently IE72 [109,110,111,112] targets these ND10 structures to quickly overcome repression and initiate a lytic infection, especially at high MOIs. 

During latent infection, however, it is likely that the same initial events occur—the genome is chromatinised immediately upon infection and that the same intrinsic immune defences are activated. However, the failure of pp71 to translocate to the nucleus and inactivate these defences upon infection of myeloid progenitors (Figure 1A) has been argued to be one possible mechanism to aid the establishment of latency [113]—although whether this is mediated solely by an interaction with hDaxx is still not clear [114]. Regardless of the exact mechanism, there is no doubt that the failure of a major viral transactivator of the MIEP (pp71) to enter the nucleus would have a significant impact upon activation of IE gene expression. Nevertheless, there are also longstanding observations that the MIEP—even after transfection—is transcriptionally less active in myeloid progenitors [77,115]. The prevailing hypothesis is that this latent phenotype is driven by the presence of high levels of transcriptional repressors in myeloid progenitors, such as YY1 and ERF, promoting long term silencing during latency via interactions with histone deacetylase and histone methyltransferase enzymes (Figure 1C). However, maybe a more refined model would suggest that levels of promoter occupancy and the recruitment of co-factors is critical to the establishment of latency rather than the absolute levels of ubiquitous transcription factors. Nevertheless, it is likely that the known interactions of YY1 and ERF with histone deacetylases and methyltransferases contribute significantly to the repressive ‘chromatin phenotype’ observed in naturally latent CD34+ haematopoietic cells around the viral MIEP [22].

It is also likely that these same mechanisms which promote silencing of the MIEP in latency are also important for driving viral latent gene expression. GATA-2 is, like YY1 and ERF, highly expressed in myeloid progenitor cells [68] and, while YY1 and ERF are binding to the MIEP mediating repression [76,79], GATA-2 binding to the LUNA and UL144 promoters drives their activity [47,54]. Interestingly, an in silico analysis of the promoters of other latently expressed genes identified putative GATA binding sites which may be important for the expression of the vIL10 and UL138 gene products. It is highly likely that histones are also recruited to latent promoters immediately post infection and, akin to the binding of ERF and YY1 to the MIEP resulting in the recruitment of histone deacetylases and histone methyltransferases, it is highly plausible that the binding of GATA-2 to the UL144 and LUNA promoters in latently infected monocytes [47] results in the recruitment of, for example histone acetyltransferases, generating the histone signature we observe in latently infected cells [46]. This ‘open’ chromatin conformation would support transcription and is consistent with viral latent gene transcription observed during HCMV latency [45,46,47]. Clearly, it remains to be seen whether GATA transcription factors are solely responsible for all the latent HCMV transcription that has been reported. Indeed, the regulation of UL138 gene expression during latency has been shown to be dependent on LUNA gene expression [55] in a long term monocyte culture model of latency [116] by, as of yet, an unidentified mechanism. However, the expression of viral genes containing binding sites for transcription factors highly expressed in the myeloid progenitor cells is highly plausible and provides further evidence for the key role cellular mechanisms of gene regulation play in the control of gene expression during persistent virus infection. 
